# The efficiency evaluation of traditional Chinese medicine hospitals by data envelopment analysis in Zhengzhou, China

**DOI:** 10.3389/fpubh.2024.1445766

**Published:** 2024-09-04

**Authors:** Jingjing Wang, Hui Lv, Hui Jiang, Wenjie Ren

**Affiliations:** ^1^Institutes of Health Central Plains, Xinxiang Medical University, Xinxiang, China; ^2^Advanced Medical & Dental Institute, Universiti Sains Malaysia, Penang, Malaysia; ^3^The First Affiliated Hospital of Xinxiang Medical University, Xinxiang, China

**Keywords:** data envelopment analysis, efficiency improvement, traditional Chinese medicine, operational efficiency, hospital efficiency

## Abstract

**Aim:**

This study aimed to evaluate the operational efficiency of traditional Chinese medicine (TCM) hospitals in China.

**Methods:**

Pearson’s analysis was used to test the correlation between the input and output variables. Data envelopment analysis (DEA) was utilized to analyze the input and output variables of 16 TCM hospitals, and each hospital efficiency score was computed by Deap 2.1, assuming variable return to scale (VRS), which is an input-oriented model. *t* tests were conducted to confirm the significant difference of efficiency scores at the hospital level and by hospital type, and ANOVA was used to test for significant differences in efficiency scores according to hospitals’ size.

**Results:**

The correlation coefficient of the input and output indicators was between 0.613 and 0.956 (*p* < 0.05). The difference in number of doctors (ND) and numbers of pharmacists (NP) were statistically significant (*p* < 0.05) at the hospital level. The mean efficiency scores for technical efficiency (TE), pure technical efficiency (PTE), and scale efficiency (SE) in secondary TCM hospitals were 0.766, 0.919, and 0.838, respectively. Additionally, the lowest TE, PTE, and SE were 0.380, 0.426, and 0.380, respectively. Eight TCM hospitals in this study were DEA efficient, with an efficiency score of 1. There were no statistically significant differences in TE, PTE, and SE among hospital levels, hospital types or hospital sizes groups (*p* > 0.05).

**Conclusion:**

This study revealed that tertiary TCM hospitals had a greater level of efficiency than secondary TCM hospitals. In our study, 50% of TCM hospitals had inefficient management. Therefore, to activate the new development power of TCM hospitals, it is necessary to reform and improve the management system and mechanism of TCM hospitals, optimize the development environment of TCM hospitals and formulate development plans and measures based on local conditions.

## Introduction

Monitoring the performance of healthcare providers is a relevant and essential worldwide issue, especially in an overwhelming demand scenario. Efficiency refers to the minimum use of inputs for a certain level of output. Countries attach great importance to the input of financial, material, and human resources into hospitals (1). Evaluating the determinants of operational efficiency is beneficial for hospital managers to design appropriate organizational strategies to address the challenges related to continuous change and decreased waste of medical resources (2, 3), such as how to effectively utilize hospital resources during the SARS-CoV-2 pandemic. The pressure on health systems may exceed their predicted capacity to handle such extreme situations (4). For instance, a study conducted in Iran suggested that the outcome of the pandemic has been impacted due to outbreak response management efficiency, determining which countries’ health systems perform better would be important by measuring the health efficiency of different countries during the COVID-19 epidemic (5).

There are several problems, including the rise in demand, the rapid growth of health expenses to incomes in developing countries and the lack of government budgets for hospitals (6). According to the World Health Report issued in 2014, 20–40% of all health sector resources are wasted (3). In China, the medical system has to maintain the health of 1.37 billion people (7, 8). Therefore, meeting the growing demand for health services in China is also a significant challenge. Previous studies conducted by different scholars have shown that the productivity and efficiency of hospitals need to be improved, especially for those secondary hospitals or those with fewer hospitals (9, 10). Effective operational evaluation benefits hospitals by identifying inefficiencies and prompting adjustments to promote their high-quality development (9). Recent research examining medical quality and operational efficiency in 57 tertiary general hospitals across China indicates a positive association between medical quality and technical efficiency (11). Additionally, another study analyzing the relationship between hospital efficiency and healthcare quality has found a positive correlation between regional hospital efficiency and healthcare quality, while the empirical evidence does not support the establishment of a causal relationship (12). By emphasizing the enhancement of healthcare service delivery and minimizing resource wastage, hospitals can attain higher levels of operational efficiency without compromising patient safety or the quality of care (13). This strategy not only promotes improved patient outcomes but also enhances the overall effectiveness and sustainability of healthcare delivery systems.

Traditional Chinese medicine hospitals (TCM hospitals), as one of the most important components of the healthcare system in China (14), have gradually established an important role in public health emergencies since the SARS-CoV-2 pandemic, such as being applied in-depth to epidemic prevention and control. In 2016, the outline of the “Healthy China 2030” emphasized that it is necessary to give full play to the characteristics of TCM, improve the service capacity of TCM and develop the health care service of TCM. In addition, the epidemic urged TCM hospitals to improve their services and emergency response capabilities, thereby allowing hospital managers to utilize medical source effectively to provide better medical services for patients. Whether TCM hospitals are public or not, it is necessary for them to use scientific and applied methods to assess the operational efficiency of the hospital for the optimal use of physical and human resources. This can be helpful in reasonably increasing hospital investment to ease the pressure of healthcare and obtain accurate information about the reasonable allocation of resources (3), thereby better maintaining or improving quality and patient safety.

Reviewing the previous literature, we found that data envelopment analysis (DEA) is a nonparametric linear programming method. It was first proposed by Farrel, and then developed by Charnes, Cooper and Rhodes (2, 3, 15, 16), who applied the CCR model to measure the technical efficiency of DMUs (decision-making units) based on the Pareto optimum concept (17). This method has been recognized as a powerful tool for performance analysis and benchmarking, spanning a wide range of industries and functional areas (18). In the medical system, it could be used to calculate the number of excess beds needed in hospitals in the case of an increase in the number of hospitalized patients during a pandemic (19), and it could also be helpful for identifying inefficient DMUs, which has a positive effect on promoting hospital improvement.

Numerous researchers from various countries have extensively utilized data envelopment analysis (DEA) to assess the efficiency of healthcare institutions in diverse contexts. Luca et al. studied the main organizational factors that generate hospital inefficiency and the internal and external features that affect hospital efficiency (2). Wang et al. identified the performance of maternal and child health hospitals in terms of productivity and efficiency (20). Yang et al. analyzed the effects of the global budgeting reimbursement system on productivity and financial efficiency by using a two-stage DEA model, thereby representing it as a key decision-making tool for hospital administrators and policymakers (17). By using DEA, Ergulen et al. estimated the effectiveness level of the Ministry of Health of the Republic of Turkey in fight of the COVID-19 epidemic, determining which months were productive or inefficient during this period (21). Mohamad et al. used this method to evaluate technical efficiency of 15 public hospitals to determine optimal hospital performance, and reported that hospital size is positively associated with technical efficiency (6). Shirouyehzad et al. created models by using DEA and MCDM methods to measure the efficiency levels of OECD countries during COVID-19 pandemic, finding six countries (Colombia, the USA, New Zealand, Denmark, and Slovakia) were efficient (5). Based on this, the purpose of this paper is to evaluate TCM hospital operational efficiency by data envelopment analysis (DEA) in Henan Province in 2020, hoping to provide appropriate advice and strategies for sustainable development of TCM hospitals in China.

## Materials and methods

### Sampling and data collection

Our study was conducted in Zhengzhou, China, which is a region located in northern China. There are 23 traditional Chinese medicine hospitals administered by the same governmental department, chosen to ensure a representative sample across various tiers of TCM healthcare facilities. We utilized a stratified proportion sampling technique to select the sampled hospitals in this study, sampling two in three facilities from ach stratum based on the type of hospital. Finally, 16 traditional Chinese hospitals were selected in this study, including four tertiary hospitals and 12 secondary hospitals. The letters represent the names of the TCM hospitals used in this study. This study was conducted in 2021, and the data in 2020 was collected for analyzing.

### Data envelopment analysis (DEA) model: input-oriented model

DEA was selected due to its suitability for evaluating the relative efficiency of traditional Chinese medicine hospitals, considering multiple input and output measures simultaneously. Instead of measuring efficiency based on averages, the DEA model is more consistent with economic theory and locates technical or Pareto inefficiencies (22), which is a method for identifying and correcting the magnitudes of these inefficiencies (23). It is widely used for the evaluation of the relative efficiency and performance of a set of decision-making units (DMUs), usually including input and output orientations. This method does not need to have assumptions for determination of the production function, and a frontier function with the internal and external factors is constructed based on the information on the inputs and outputs of DMUs (5). For example, when hospital managers intend to more readily control the resources used for patient treatments (18) and focus on minimizing the use of inputs to produce a given output (24), a better choice is to adapt an input-oriented model. However, DMUs are given a fixed quantity of resources (inputs) and are asked to maximize output in the output-orientated DEA (25). In addition, we can estimate whether each hospital is efficient depending on its efficiency value, meaning that units with a score of 1 are considered effective, whereas other units with efficiency scores below 1 are called inefficient units (26). Specifically, we would identify related variables, like inputs variables (e.g., resources, staff) and outputs variables (e.g., Number of outpatient, bed utilization) and quantified for each hospital included in the analysis.

Researchers tend to choose suitable DEA models depending on their research purpose. For instance, the BCC model can be used to evaluate the relative efficiency of minimal input consumption for a given level of outputs, or the increase in output for a given input (17, 23). Many hospital providers are expected to provide a given level of healthcare services with the maximal resources. In other words, the primary purpose of analyzing hospital efficiency is to identify inefficient aspects that decrease hospital costs. Therefore, according to the aim of this study, an input-oriented model was used in this study, and a BCC model with variable-scale returns was chosen.

### Input–output variables selection

Before starting this study, we reviewed the related literature related to hospital efficiency to acquire input–output variables, thereby providing good knowledge about what variables were essential and applied in existing studies. We found that the input variables included capital (e.g., equipment, hospital beds, etc.), land (e.g., area), labor (e.g., physician, nursing staff, etc.) and others; the outputs included patient, bed utilization, service and economic output. All of these factors are shown in Table 1.

**Table 1:** Factors affecting hospital efficiency.

Inputs		Outputs	
Capital	Total beds (9, 14, 19, 26, 28, 35, 38, 39)	Patients	Number of outpatient or/and inpatient cases (6, 9, 10, 18, 30, 38)
	Number of existing beds (1, 29, 31)		Number of discharged patients (1, 3, 10, 14, 18, 26, 28–31, 34)
	Number of opening beds (1, 3, 6, 8, 10, 30, 34)		Number of outpatient and emergency patients (8, 31)
	Number of devices over 10,000 Chinese Yuan (1, 34)		Cured cases and improved cases (1, 39)
land	Structure/building/hospital area (1, 9, 34, 35)		Outpatient or/and emergency visits (1–3, 10, 26, 35)
Labor	Number of total staffs (1, 3, 6, 9)	Bed utilization	Outpatients per day (35)
	Number of health workers (1, 28, 29, 31, 39)		Days of hospital bed turnover (1)
	Number of doctors/physicians (1, 8, 10, 18, 19, 26, 30, 34, 35, 38)		Turnover times (1, 3)
	Number of TCM personnel (14)		Inpatient days/average length of stay (3, 6, 8, 26, 34, 35)
	Number of nurses (2, 8, 10, 18, 19, 26, 30, 34, 35, 38)		Working days of hospital beds (1)
	Pharmacists of western medicine (1)		Bed occupancy/utilization rate (1, 2, 19, 28, 30)
	Numbers of pharmacists (38)	Service	Number of health examinations (39)
	Numbers of other personnel (administrative, technical staff and logistic staff) (1, 2, 8, 19, 26, 35, 38)		Number of surgeries (6, 18, 26)
Others	Total expenditure (10, 30)	Others	Annual revenues of hospitals (26)
	Total assets (1, 30)/operating cost (2, 26)/subsidies (1)/total public funding (in dollar) (39)/total public funding (in dollar) (39)		Business revenue (1, 2)

The number of DMUs (Decision Making Units) should be double the total count of inputs and outputs combined, as argued by Dyson et al. (27). To meet this constraint and data accessibility, 8 variables were selected for the current study, as shown in Table 2. Some scholars have reported that Pearson analysis can be useful for measuring the significance of the linear correlation between input and output variables to test isotonicity (27–29). Before using this method, a homogeneity test of variance was performed by the Kolmogorov–Smirnov test, and all *p* values were over 0.05, indicating that Pearson analysis can be used to test the correlation between these input and output variables, and these results revealed strong correlations among them in this study. The correlation coefficients of the variables included in this study were shown in Table 2. Descriptive statistics of the inputs and outputs used in the analyses are presented in Table 3.

**Table 2:** Correlation analysis of variables for input and output.

Inputs	Outputs	
	Number of outpatient and emergency patients (NOEP)	Number of discharged patients (NDP)
Number of opening beds (NOP)	0.865^**^	0.956^**^
Number of doctors (ND)	0.883^**^	0.899^**^
Number of TCM personnel (NTCMP)	0.923^**^	0.826^**^
Number of nurses (NN)	0.869^**^	0.928^**^
Numbers of pharmacists (NP)	0.850^**^	0.791^**^
Building area (BA)	0.613^*^	0.695^**^

^*^p < 0.05, ^**^p < 0.01.

**Table 3:** Descriptive statistics for the sample TCM hospitals.

Code	Explanation of the variable	Tertiary hospital			Secondary hospital		
		*N*	Mean	SD	*N*	Mean	SD
Input variables							
NOB	Number of opening beds	4	691.750	439.643	12	294.83	318.181
ND	Number of doctors	4	260.250	199.326	12	91.250	93.522
NTCMP	Number of TCM personnel	4	139.500	126.221	12	55.500	55.841
NN	Number of nurses	4	411.500	357.501	12	154.750	164.130
NP	Numbers of pharmacists	4	40.250	20.694	12	14.000	12.563
BA	Building area	4	41,803.750	22,215.957	12	26,461.417	33,612.814
Output variables							
AOEP	Number of outpatient and emergency patients	4	297,998.000	368,206.877	12	98,696.667	120,539.210
NNP	Number of discharged patients	4	19,988.000	13,157.814	12	8,276.000	11,712.093

### Data analysis

First, Pearson analysis was used to test the correlation between the input and output variables to determine which variables could be chosen in this study. Then, DEA was used to analyze the input–output variables of all TCM hospitals that we selected, and each hospital efficiency score was computed by Deap 2.1, assuming variables return to scale (VRS), which is an input-oriented model. Previous studies mentioned that hospital characteristics (including size and ownership type) influence efficiency (6, 18, 26). TCM hospital ownership types were divided in our study, including public and nonpublic. According to a previous study, we found that there were three types of hospital sizes: small (fewer than 500 beds), medium (500–1,000 beds) and large (more than 1,000 beds) (18), the same method of division was used to determine hospital size in this study. In this study, ANOVA *F*-tests or t tests were conducted to confirm the significant differences in TCM hospital ownership type and hospital size.

### Ethics

The participants in this study did not focus on human beings, and there was no relationship between the data collected and the patients’ medical records. Therefore, an ethics statement was not needed.

## Results

### Correlation analysis

According to correlation analysis, we found that all correlation coefficients were greater than 0.600, indicating that there was a strong positive correlation between the input and output variables (*p* < 0.05). The correlation coefficient of the input–output indicators was between 0.613 and 0.956, as shown in Table 2.

### Basic characteristics of the sample TCM hospitals

Sixteen TCM hospitals were selected in this study, including four tertiary hospitals and 12 secondary hospitals. The variables for the inputs and outputs are shown in Table 3. For tertiary hospitals, the average numbers of opening beds and building areas were 691.750 and 41,803.750 m^2^, respectively. The average numbers of health workers were doctors (260.250), TCM personnel (139.500), nurses (411.500) and pharmacists (40.250), respectively. For secondary hospitals, the average number of outpatient and emergency patients was 297,998.000, and the average number of discharged patients was 19,988.000. Comparing the variables of inputs and outputs between tertiary hospitals and secondary hospitals, the difference in ND and NP was statistically significant (*p* < 0.05), and there was no significant difference in the other six items (*p* > 0.05).

### Operational efficiency of TCM hospitals

DEA-BCC was conducted for the two data groups. The letters represent the names of the TCM hospitals, and the results are presented in Table 4. A–D were used to represent four tertiary TCM hospitals, including three general public hospitals and one specialized non-public hospital, and all of these TCM hospitals had an efficiency score of 1, meaning that these hospitals were on the efficient production frontier.

**Table 4:** The scores of the efficiency of TCM hospitals by DEA.

Hospital type		DMUs	TE	PTE	SE	RS
Tertiary TCM hospital	Public	A	**1.000**	**1.000**	**1.000**	–
		B	**1.000**	**1.000**	**1.000**	–
		C	**1.000**	**1.000**	**1.000**	–
	No-public	D	**1.000**	**1.000**	**1.000**	–
Secondary TCM hospital	Public	E	0.386	0.426	0.905	irs
		F	**1.000**	**1.000**	**1.000**	–
		G	0.697	0.700	0.995	irs
		H	0.480	0.904	0.531	irs
	No-public	I	**1.000**	**1.000**	**1.000**	–
		J	0.380	1.000	0.380	irs
		K	0.855	1.000	0.855	irs
		L	0.900	1.000	0.900	irs
		M	0.935	1.000	0.935	irs
		N	**1.000**	**1.000**	**1.000**	–
		O	0.560	1.000	0.560	irs
		P	**1.000**	**1.000**	**1.000**	–

DUMs, Decision making units; TE, Technical efficiency; PTE, Pure technical efficiency; SE, Scale efficiency. RS, Return to scale; irs: Increasing; –: constant. The meaning of bold values suggested these hospitals were on the efficient production frontier.

E–P represents 12 secondary TCM hospitals, including four general public hospitals, seven general non-public hospitals and one specialized non-public hospitals. The mean efficiency scores of TE, PTE, and SE in secondary TCM hospitals were 0.766, 0.919, and 0.838, respectively. Additionally, among TCM secondary hospitals, we found that only four hospitals acquired efficient scores, with an efficiency score of 1. Two hospitals had efficiency scores ranging from 0.8 to 1, representing weekly efficiency. Besides, three hospitals had efficiency scores lower than 0.5, which here classified as extremely inefficient. The lowest TE, PTE, and SE efficiency scores were 0.380, 0.426, and 0.380, respectively.

Eight TCM hospitals (A, B, C, D, F, I, N, and P) were DEA efficient, and the returns to scale were constant (−), meaning that the development of these hospitals entered a stable period. Maintaining the dynamic balance of input and output can guarantee their stable development. In secondary hospitals, the return to scale of 8 hospitals (E, G, H, J, K, L, M, and O) increased (inputs should be increased to improve efficiency). Eight TCM hospitals had constant returns to scale (changes in the number of inputs did not affect efficiency), and 50% of TCM hospitals showed an increase in returns to scale.

### The differences in hospital efficiency scores according to hospital characteristics

According to the *t* test and *F* test, we found that there were no statistically significant differences in TE, PTE, and SE among hospital levels, hospital types or hospital sizes groups (*p* > 0.05), as shown in Table 5.

**Table 5:** Differences in hospital efficiency scores according to hospital characteristics.

		Number of hospitals (*n*)	TE	PTE	SE
Hospital level	Tertiary TCM hospital	4	1 (0.000)	1 (0.000)	1 (0.000)
	Secondary TCM hospital	12	0.767 (0.251)	0.919 (0.178)	0.838 (0.219)
	*t*		1.817	0.885	1.440
	*p* value		0.091	0.391	0.172
Ownership type	Public	7	0.795 (0.272)	0.861 (0.221)	0.918 (0.175)
	Non-public	9	0.848 (0.225)	1 (0.000)	0.848 (0.225)
	*t*		−0.428	−1.897	0.687
	*p* value		0.675	0.079	0.489
Hospital size	Less than 500 beds	2	0.811 (0.242)	0.990 (0.030)	0.816 (0.235)
	500–1,000 beds	4	0.771 (0.294)	0.782 (0.276)	0.975 (0.047)
	More than 1,000 beds	10	1 (0.000)	1 (0.000)	1 (0.000)
	*F*		0.621	3.653	1.370
	*p* value		0.553	0.055	0.288

TE, Technical efficiency; PTE, Pure technical efficiency; SE, Scale efficiency.

## Discussion

TCM hospitals play an important role in health systems, and it is essential for health providers to maintain sustainable development. Especially during the pandemic, demand for health services increased, and TCM hospitals were forced to increase the capacity of their resources owing to pressure on the health system, such as the number of hospital beds and health workers available for handling such a special situation. Evaluating TCM operational efficiency can be helpful in identifying inefficiency sources, thereby making it possible to reduce hospital costs and decrease wastage of hospital sources. On the other hand, the optimal utilization of hospital capacity and inputs is more efficient than building new hospitals to meet the needs of patients (19). According to our analysis of the efficiency of TCM hospitals during the pandemic, knowledge of their operational status can be used to identify inefficiency parts in helping hospitals to effectively allocate resources.

Comparing the input and output variables between tertiary hospitals and secondary hospitals, revealed that the difference in the number of doctors and pharmacists was statistically significant (*p* < 0.05). Additionally, we found that the average numbers of doctors and pharmacists in tertiary TCM hospitals were 260.250 and 40.250, respectively, which were much higher than the average numbers in secondary hospitals (91.250 and 14.000, respectively). A previous study reported that there was a statistically significant difference in the number of outpatient visits to tertiary hospitals (*p* < 0.05) (9), while we found that the difference in the number of outpatient and emergency patients was not statistically significant (*p* > 0.05). By using BCC-DEA, we found that all tertiary TCM hospitals in our study had an efficiency score of 1, which was in line with the findings of Li et al. (9). 50% of TCM hospitals had inefficient management in our study, which was higher than that reported in an Iranian study conducted by Soroush et al. (19) and lower than the findings of in other studies (2, 3, 6, 30, 31). A study has shown a different result that the overall effective rate of secondary public hospitals (30.77%) was higher that of tertiary public hospitals (19.44%) (32). Moreover, a study conducted by Li in China revealed that the mean efficiency scores of TE, PTE, and SE among TCM hospitals in China were 0.870, 0.911, and 0.957 (29), which were higher than the mean efficiency scores of TE and SE of secondary TCM hospitals in our study, suggesting that the hospitals in our study operated at low efficiency. Meanwhile, these results reflected that tertiary hospitals have a greater level of efficiency than secondary hospitals, which was consistent with the results of some studies (9, 33). Additionally, three secondary TCM hospitals had efficiency scores lower than 0.5 in our study, and TEs and SEs among them had the lowest scores, meaning that this TCM hospital had a severely inefficient technical level. This can be explained by the fact that tertiary hospitals have stronger superiority than secondary hospitals in local policy financial investment, medical capacity, and competition for patient resources. Under this poor background, it is necessary to seek for some ways for improving comprehensive ability of secondary hospitals, such as signing contracts with large hospitals, forming specialty alliances or medical alliances, thereby making it possible to strength their operational efficiency. Besides, the results had also indicated considerable room for TE improvement in secondary TCM hospitals. Such measures like procurement of low-cost and high-quality medical equipment and supplies could be referenced for improving efficiency of hospitals.

The difference in efficiency between secondary and tertiary hospitals mainly came from differences in the management level of hospital managers and the technical level of doctors. For example, previous literature revealed that the proportion of health professionals had a positive association with efficiency scores (25). Some scholars believe that medical personnel prefer to work in high-level hospitals for better conditions, higher salaries, and bright development prospects (9, 25). Moreover, the national policy of emphasizing western medicine and neglecting TCM may be a key factor that leads to a deterioration in the development of TCM hospitals (9). Therefore, as the main providers of TCM, TCM hospitals should consider corresponding policies and measures to improve their efficiency for stable development, especially for secondary TCM hospitals. For example, hospitals can optimize the personnel structure by increasing healthcare workers with high-quality and reducing these employees with less-skilled.

Previous studies have shown that TCM hospitals with low PTE should focus on improving hospital management and decision-making, and TCM hospitals with low SE should emphasize appropriately carrying out scale operation construction as their keystone (31, 34) Additionally, the efficiency of TCM hospitals is a great way to determine information related to the state of hospital operation, which is beneficial for making reasonable decisions for the development of hospitals. These measures, such as learning up-to-date management system concepts, optimizing hospital operation scales, and effective allocation, can be important for improving the technological capability and management ability of hospitals.

Comparing DEA scores at different hospital levels can be helpful for explaining, to some extent, the development of medical and health resources at different levels. Analysis of the differences in TE, PTE, and SE between secondary and tertiary TCM hospitals revealed that there was no significant difference in the level of hospitals, which was inconsistent with previous research showing that the difference in PTE between secondary and tertiary hospitals was statistically significant (9). Mohamad et al. (6) reported similar results using Tobit regression. We found that hospital type was a nonsignificant driver of efficiency in our study, which was in line with the findings of Luca et al. (2) and Xing et al. (35) In addition, we found that there was no significant difference between hospital size and efficiency scores in this study, which does not align with the findings that increasing the number of hospital beds could improve the TE value of hospitals with different bed sizes (31, 36) or that the size of the hospital had a significant effect on hospital efficiency (2, 6, 37). The findings of a study on the efficiency assessment of tertiary hospitals in China showed that the suitable number of beds in the hospital was 1,001–2,000 beds (31).

In analyzing hospital efficiency, different scholars intend to choose different indicators, making it possible to obtain different results. There were several limitations in this study. First, TCM hospitals, which are representative of hospitals in that city, were evaluated for only 1 year in this study. Second, some environmental factors that affect the efficiency of TCM hospitals, such as local economic level, and health service costs, had not been analyzed. Third, the selected variables may not be perfect, although we reviewed indicators related to hospital efficiency according to previous literature. Fourth, we were unable to assess the role of environmental factors such as population size and poverty due to a lack of available data. Therefore, further research on TCM hospitals in other regions is needed to better understand the efficiency of TCM hospitals in China. In addition, the indicators did not exhibit significant differentiation between traditional Chinese medicine (TCM) hospitals and Western medicine hospitals. Furthermore, we aim to investigate prospective strategies aimed at bolstering their resilience amidst these evolving circumstances in forthcoming researches.

## Conclusion

This study measured the TE, PTE, and SE of municipal TCM hospitals by using the DEA-BCC model, the main finding of which was tertiary TCM hospitals have a higher level of efficiency than secondary TCM hospitals. In our study, 50% of TCM hospitals had inefficient management. In addition, there were no statistically significant differences in TE, PTE, and SE among hospital levels, hospital types or hospital sizes groups (*p* > 0.05). Therefore, to activate the new development power of TCM hospitals, it is necessary to reform and improve the management system and mechanism of TCM hospitals, optimize the development environment of TCM hospitals and formulate development plans and measures based on local conditions.

## Data Availability

The raw data supporting the conclusions of this article will be made available by the authors, without undue reservation.
